# Deciphering Multidrug-Resistant Acinetobacter baumannii from a Pediatric Cancer Hospital in Egypt

**DOI:** 10.1128/mSphere.00725-21

**Published:** 2021-11-17

**Authors:** Deena Jalal, Mariam G. Elzayat, Aya A. Diab, Hend E. El-Shqanqery, Omar Samir, Usama Bakry, Reem Hassan, Mervat Elanany, Lobna Shalaby, Ahmed A. Sayed

**Affiliations:** a Genomics Program, Children’s Cancer Hospital Egypt 57357, Cairo, Egypt; b Department of Clinical Pathology, Faculty of Medicine, Cairo University, Cairo, Egypt; c Molecular Microbiology Unit, Children’s Cancer Hospital Egypt 57357, Cairo, Egypt; d Microbiology Unit, Children’s Cancer Hospital Egypt 57357, Cairo, Egypt; e Infectious Disease Unit, Children’s Cancer Hospital Egypt 57357, Cairo, Egypt; f Department of Pediatric Oncology, National Cancer Institute, Cairo University, Cairo, Egypt; g Department of Biochemistry, Faculty of Science, Ain Shams University, Cairo, Egypt; University of Nebraska Medical Center

**Keywords:** *Acinetobacter baumannii*, antimicrobial resistance genes, whole-genome sequencing

## Abstract

Infection by multidrug-resistant (MDR) Acinetobacter baumannii is one of the major causes of hospital-acquired infections worldwide. The ability of A. baumannii to survive in adverse conditions as well as its extensive antimicrobial resistance make it one of the most difficult to treat pathogens associated with high mortality rates. The aim of this study was to investigate MDR A. baumannii that has spread among pediatric cancer patients in the Children’s Cancer Hospital Egypt 57357. Whole-genome sequencing was used to characterize 31 MDR A. baumannii clinical isolates. Phenotypically, the isolates were MDR, with four isolates showing resistance to the last-resort antibiotic colistin. Multilocus sequence typing showed the presence of eight clonal groups, two of which were previously reported to cause outbreaks in Egypt, and one novel sequence type (ST), Oxf-ST2246. Identification of the circulating plasmids showed the presence of two plasmid lineages in the isolates, strongly governed by sequence type. A large number of antimicrobial genes with a range of resistance mechanisms were detected in the isolates, including β-lactamases and antibiotic efflux pumps. Analysis of insertion sequences (ISs) revealed the presence of IS*Aba1* and IS*Aba125* in all the samples, which amplify β-lactamase expression, causing extensive carbapenem resistance. Mutation analysis was used to decipher underlying mutations responsible for colistin resistance and revealed novel mutations in several outer membrane proteins, in addition to previously reported mutations in *pmrB*. Altogether, understanding the transmissibility of A. baumannii as well as its resistance and virulence mechanisms will help develop novel treatment options for better management of hospital-acquired infections.

**IMPORTANCE** Acinetobacter baumannii represents a major health threat, in particular among immunocompromised cancer patients. The rise in carbapenem-resistant A. baumannii, and the development of resistance to the last-resort antimicrobial agent colistin, complicates the management of A. baumannii outbreaks and increases mortality rates. Here, we investigate 31 multidrug resistant A. baumannii isolates from pediatric cancer patients in Children’s Cancer Hospital Egypt (CCHE) 57357 via whole-genome sequencing. Multilocus sequence typing (MLST) showed the presence of eight clonal groups including a novel sequence type. *In silico* detection of antimicrobial-resistant genes and virulence factors revealed a strong correlation between certain virulence genes and mortality as well as several point mutations in outer membrane proteins contributing to colistin resistance. Detection of CRISPR/Cas sequences in the majority of the samples was strongly correlated with the presence of prophage sequences and associated with failure of bacteriophage therapy. Altogether, understanding the genetic makeup of circulating A. baumannii is essential for better management of outbreaks.

## INTRODUCTION

Acinetobacter baumannii has been recognized as one of the major causes of hospital-acquired infections (1, 2). Its spectrum of illness is wide and includes bloodstream infection, pneumonia, and endocarditis (3). A. baumannii has a remarkable capacity to develop antimicrobial resistance, which is largely related to mobile genetic elements, such as insertion sequences, plasmids, and antibiotic resistance islands (1). The rise in carbapenem-resistant A. baumannii (CRAB) represents a major health threat, as it prohibits the use of the broad-spectrum carbapenem antibiotics that are reserved for the treatment of severe infections in hospitalized patients. The major mechanism of carbapenem resistance in A. baumannii is through the production of class B metallo-β-lactamases such as *bla*_NDM,_ *bla*_VIM_, *bla*_IMP_, and *bla*_SIM_ (4, 5), as well as carbapenem-hydrolyzing class D β-lactamases (CHDLs), in particular subgroups *bla*_OXA-23_, bla_OXA-24_, *bla*_OXA-51_, and *bla*_OXA-68_ (6–8).

The prevalence of CRAB in Egypt is the highest in the Middle East (9), in particular among immunocompromised and intensive care unit (ICU) patients (10, 11). Several studies have investigated the dissemination of carbapenemases such as *bla*_NDM_, *bla*_OXA-51-like_, and *bla*_OXA-23-like_ in A. baumannii and have reported a prevalence of more than 70% among isolates in Egypt (12, 13). The propagation of carbapenemase-encoding genes is in large part due to their association with integrons and plasmids (14, 15), allowing their rapid transmission and accumulation. Insertion sequences (ISs) in the region surrounding these genes cause their overexpression by providing promoter sequences, resulting in higher MICs for carbapenems (16–20).

The cationic peptides polymyxins B and E (colistin) have been available for clinical use since the 1950s due to their effectiveness against Gram-negative bacteria (21). Systemic toxicity due to the administration of polymyxin B, and to a lesser extent colistin, led to their replacement by other antimicrobial drugs (21, 22). The recent prevalence of extensively drug-resistant pathogens such as A. baumannii caused the reemergence of polymyxins as last-resort antibiotic therapy. Polymyxins exert their antimicrobial activity by binding to the lipopolysaccharide (LPS) layer on the outer membrane of Gram-negative bacteria, causing it to swell, which subsequently disrupts the osmotic balance of the cell, causing its death (22). Although still uncommon, the increasing use of colistin caused the emergence of colistin-resistant (ColR) strains, increasing the incidence of pan-drug-resistant pathogens, which are resistant to all available antimicrobials (23). Reports of ColR strains emerging in Egypt have surfaced in the past few years, complicating the management of CRAB infections further and increasing mortality rates (12, 24).

The ability of A. baumannii to develop multidrug resistance, survive on inanimate surfaces, and resist serum killing, as well as its aggressiveness in host epithelial cell invasion makes it one of the most important enemies in intensive care units, being the cause of many outbreaks (1, 25). A. baumannii owes its remarkable aggressiveness and tenacity to an impressive repertoire of virulence genes. Outer membrane porins, such as OmpA, represent a major virulence factor that allows for adherence to and invasion of host epithelial cells (26). Biofilm formation, which depends on AdeFGH efflux pumps, biofilm-associated protein (Bap), Csu fimbriae, poly-β-1,6-*N-*acetylglucosamine (PNAG) surface polysaccharides, pili, and OmpA, underlies the ability of A. baumannii to survive on abiotic surfaces, allowing it to persist and spread in the hospital environment (27). Capsular and lipopolysaccharides (LPS) in the cell wall of A. baumannii modulate the bacterial resistance to peptide antibiotics (28), evasion of the immune response, and thus, serum resistance (29). Phospholipases, porins, and proteases secreted through outer membrane vesicles (OMVs) aid in the invasion of host epithelial cells (30, 31).

Different methods have been used to investigate outbreaks of A. baumannii within hospitals, such as pulsed-field gel electrophoresis (PFGE) and multilocus sequencing typing (MLST) (32). However, such techniques have important limitations for source tracking of infections in hospitals, as they examine specific sites in the genome, which may result in false clustering of unrelated strains (33). In the past decade, whole-genome sequencing (WGS) has been used to investigate hospital outbreaks using ultrahigh resolution, thus discriminating two isolates differing by just a single mutation (34, 35). Benchtop sequencing instruments now offer a cost-effective approach for bringing bacterial WGS to the clinical environment (36).

The need for new antimicrobials has never been more compelling. Understanding the modes of bacterial resistance and the development of novel antimicrobials is urgently required. In this paper, we utilized next-generation sequencing technology to investigate 31 A. baumannii isolates from clinical samples of 27 hospitalized patients in a tertiary care hospital through whole-genome sequence comparisons and phylogenetic analyses. This was followed by *in silico* detection of the A. baumannii resistomes and virulomes, as well as CRISPR/Cas and phage sequences. Discerning the genetic elements encompassed in the A. baumannii genome would aid in understanding the transmissibility of the bacteria and the development of prospective treatment options.

## RESULTS

### Clinical characteristics.

A total of 31 isolates of A. baumannii were obtained from 27 infected pediatric cancer patients with different types of malignancies from Children’s Cancer Hospital Egypt (CCHE) 57357. The recovery rate was 22% (*n *= 7) and was determined if negative A. baumannii cultures were obtained after treatment. Phenotypic characterization of all 31 isolates by antibiotic susceptibility testing (AST) showed multidrug resistance. Isolates were considered multidrug-resistant (MDR) if they were nonsusceptible to at least one agent in three or more antimicrobial categories and extensively drug-resistant (XDR) if they were nonsusceptible to all but two or fewer antimicrobial classes (23).

### Sequence typing.

**FIG 1:**
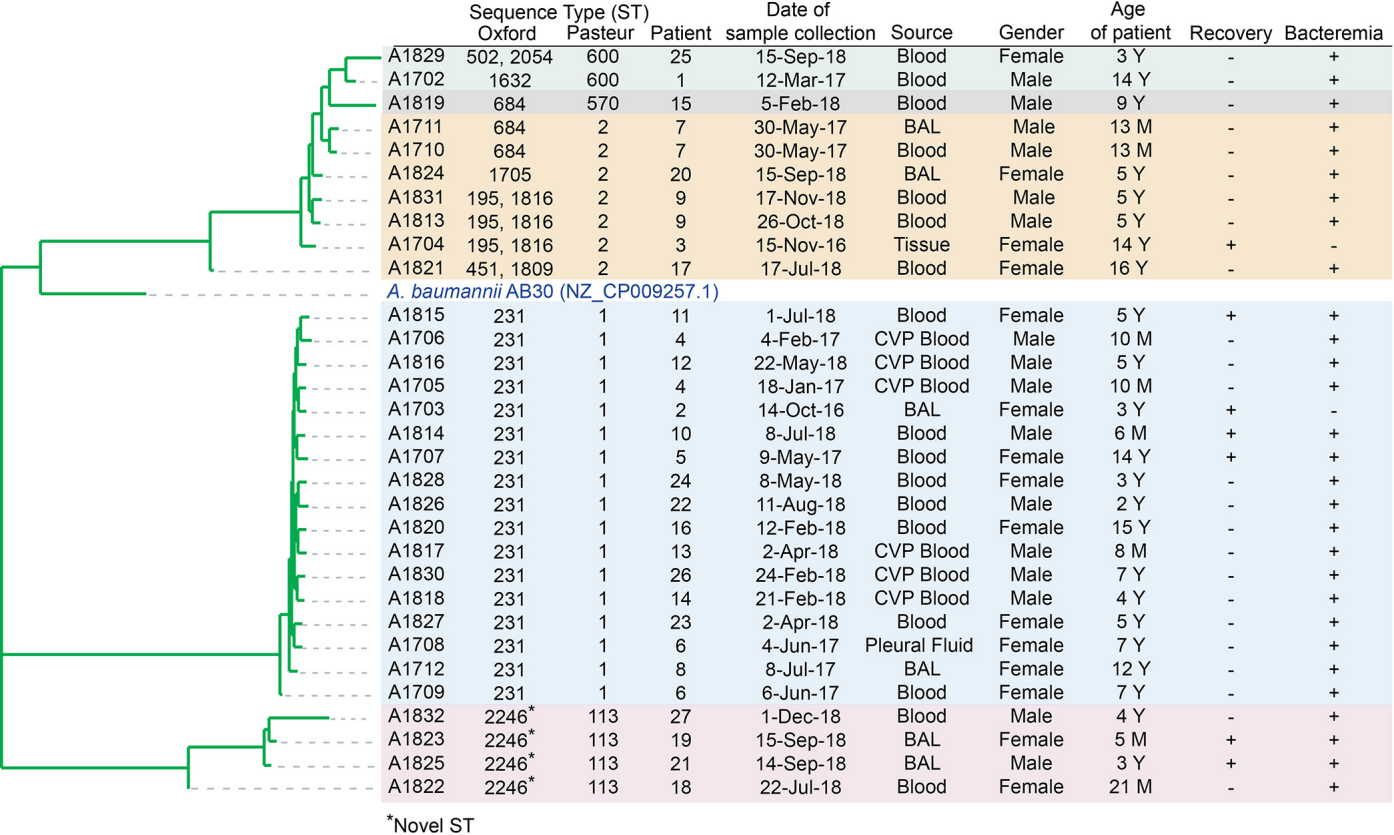
Phylogenetic tree and demographic data. Merged reads were mapped to the A. baumannii reference (GenBank accession number NZ_CP009257.1) to create a phylogenetic tree. The similarity distance between the isolates was analyzed based on SNP analysis and appended to a table showing the clinical data of the patients, and the sequence type of the isolated A. baumannii is appended. Recovery, + for negative cultures after treatment, – for persistent positive cultures until death of patient; BAL, bronchioalveolar lavage; CVP, central venous port. Isolates are color-shaded based on Pasteur ST.

*In silico* MLST analysis, using the Oxford scheme, revealed the presence of eight groups, Oxf-ST231, Oxf-ST1632, Oxf-ST684, Oxf-ST1705, Oxf-ST195, Oxf-ST451, and Oxf-ST502 and one novel ST, designated Oxf-ST2246. The Oxford scheme detected an alternative *gdhB* locus (*gdhB2*) in five isolates, which was classified as *gdhB* allele 189 in addition to the allele of the proper *gdhB* locus (37). This led to the detection of two sequence types (STs) in these five isolates, Oxf-ST2054 and Oxf-ST502 in A1829, Oxf-ST1816 and Oxf-ST195 in A1831, A1813, and A1704, and Oxf-ST1809 and to Oxf-ST451 in A1821 (Fig. 1). On the other hand, the Pasteur scheme revealed five groups of STs, Pas-ST1, Pas-ST2, Pas-ST570, Pas-ST600, and Pas-ST113. This is expected, given that the Oxford MLST scheme is more capable at differentiating between closely related isolates (37). However, due to the *gdhB* paralogy in the Oxford scheme, which complicates ST detection, the Pasteur scheme will be used here. The phylogenetic relationship between different isolates is shown in Fig. 1.

### Plasmid distribution in the different isolates.

**FIG 2:**
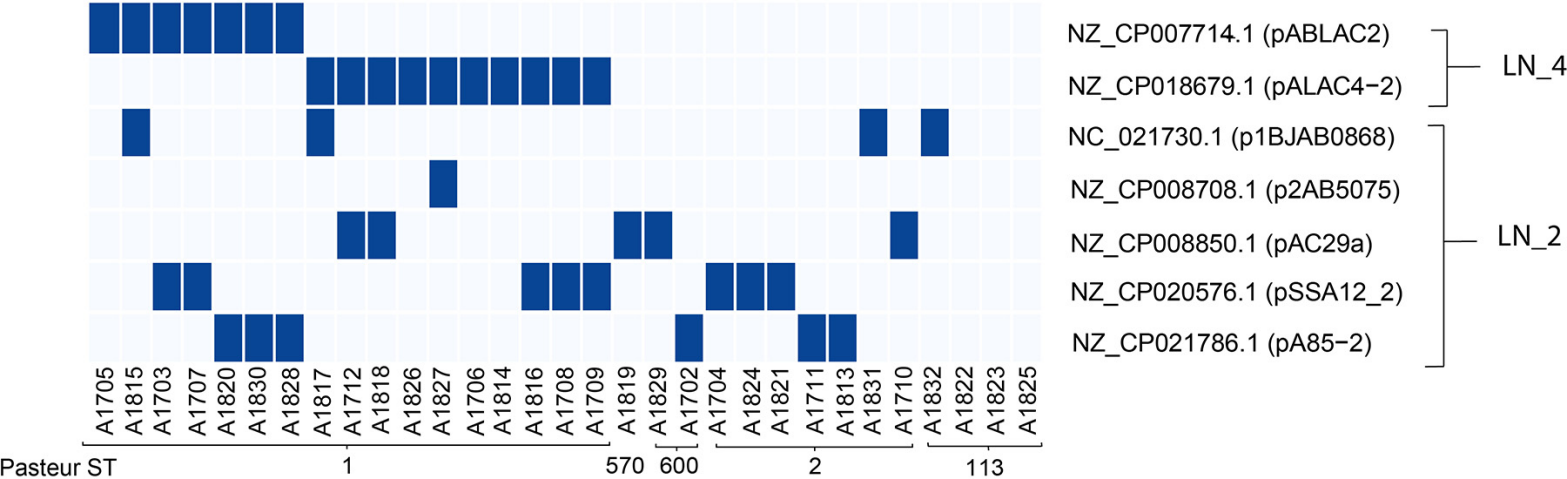
Heat map showing the presence or absence of each plasmid in each sample. Dark blue shows the presence of the plasmid, while light blue indicates its absence.

Seven plasmids were detected in the isolates, belonging to two different lineages (LN_2 and LN_4) (38; Fig. 2). Five plasmids (p1BJAB0868, p2AB5075, pAC29a, pSSA12_2, and pA85-2) belong to LN_2, and share 99.8% sequence homology. The other two plasmids (pABLAC2 and pALAC4-2) belong to LN_4 and share 100% sequence identity. The distribution of pABLAC2 and pALAC4-2 is confined to isolates of ST1.

### Antibiotic resistance profile.

**FIG 3:**
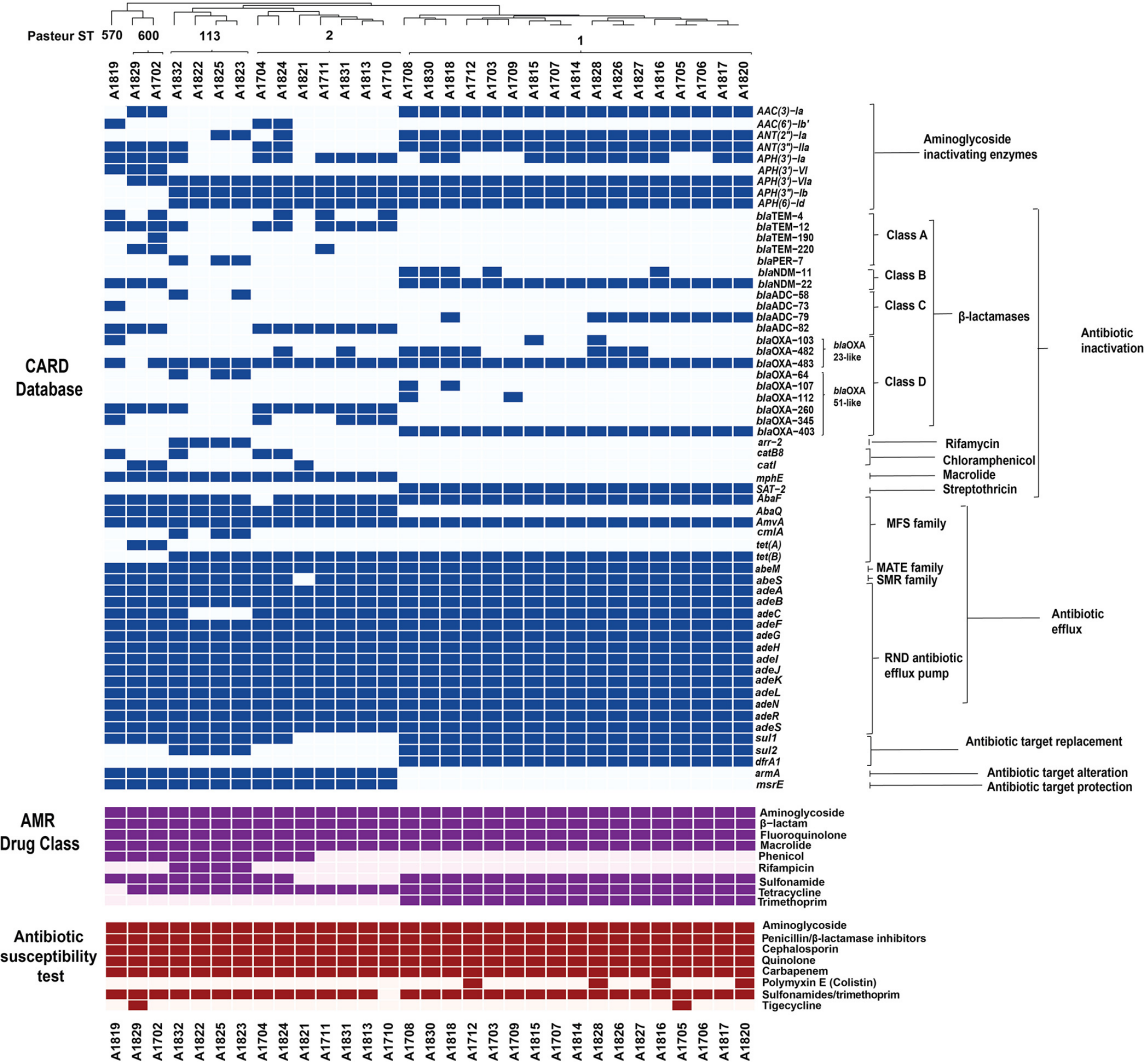
Complex heat maps showing the presence or absence of each antibiotic resistance gene and antibiotic resistance drug class and the observed antibiotic susceptibility. The antibiotic resistance genes detected are shown in blue; dark blue shows the presence of the antibiotic resistance gene, while light blue indicates its absence. The antibiotic classes for which the detected genes confer resistance are shown in purple; dark purple shows expected resistance to a drug class, while light purple shows susceptibility. The phenotypic antibiotic resistance based on AST is shown in maroon; dark maroon shows resistance to the antibiotic, while light maroon shows susceptibility.

AST was performed on the 31 A. baumannii isolates, and all of them were found to be multidrug-resistant (23; and Fig. 3). Almost all isolates were resistant to the sulfonamide-trimethoprim combination, except for isolate A1710. On the other hand, most isolates were susceptible to colistin (except for isolates A1712, A1816, A1820, and A1828) and tigecycline (except for isolates A1705 and A1829).

### Antibiotic resistance genes and mobile genetic elements.

**FIG 4:**
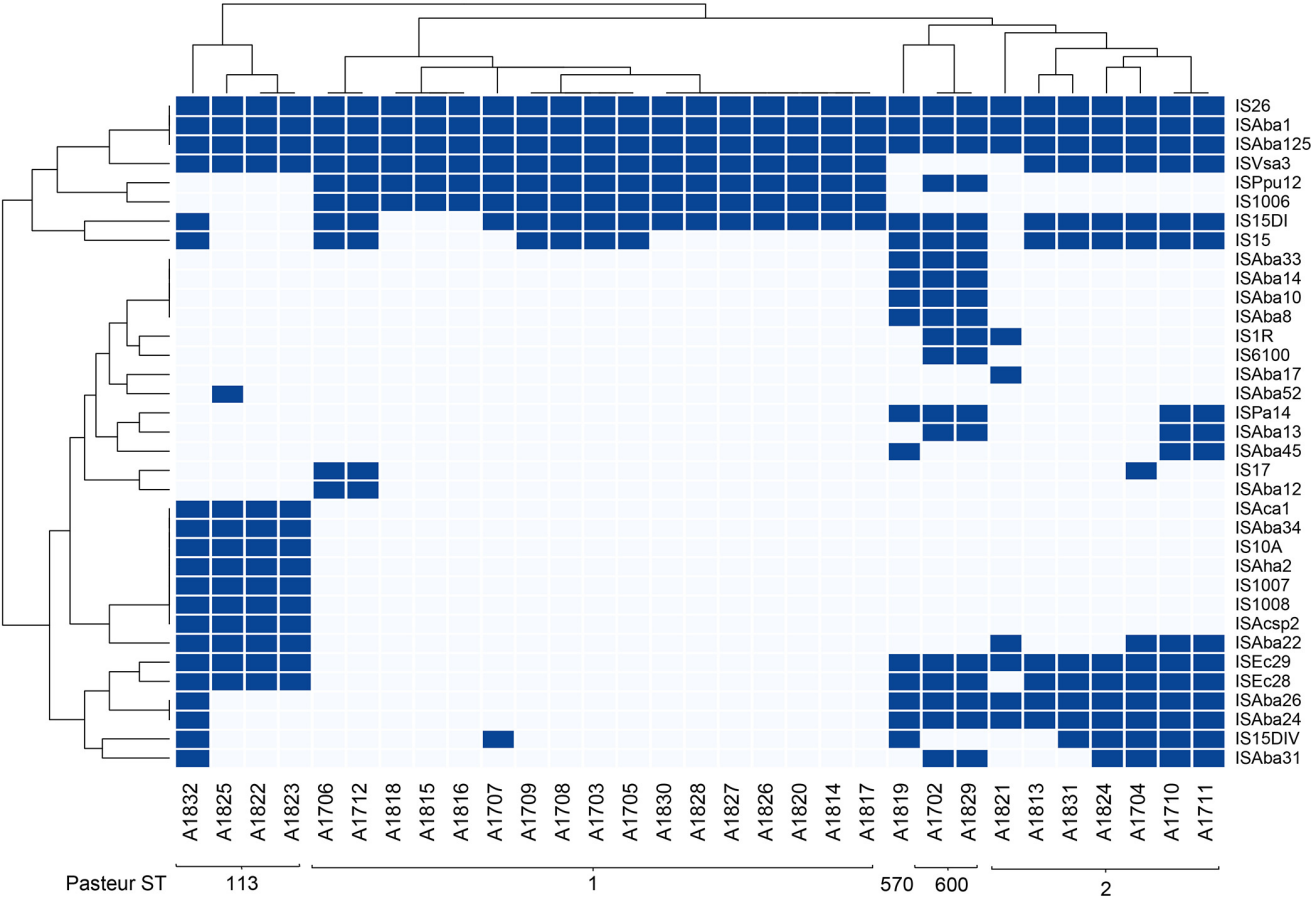
Heat map showing the presence or absence of each IS. Dark blue shows the presence of the IS, while light blue indicates its absence.

**FIG 5:**
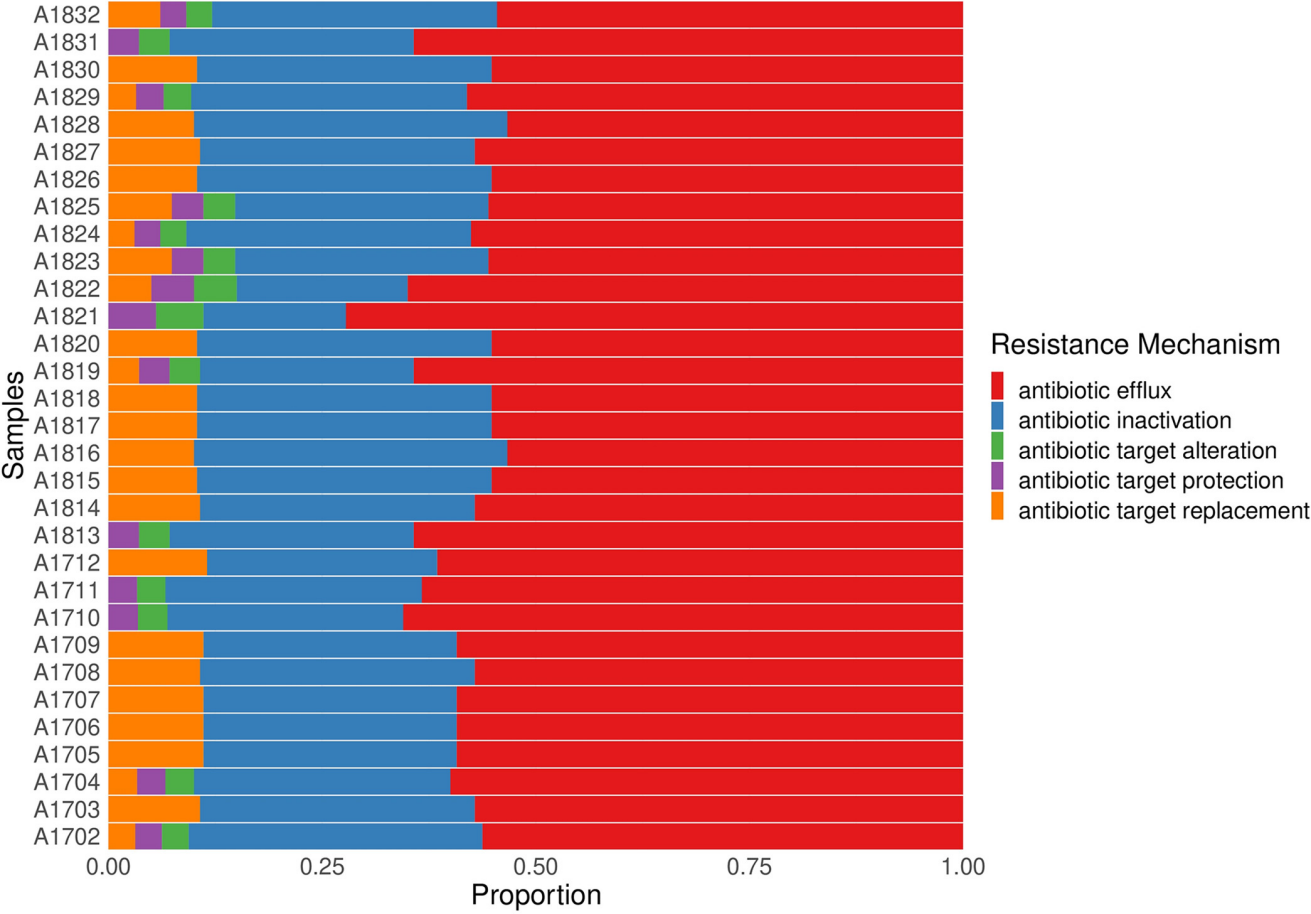
Stacked bar graph showing the resistance mechanisms represented in each isolate. After grouping of the antibiotic resistance genes by mechanism. Each color represents a mechanism of resistance; red, antibiotic efflux; blue, antibiotic inactivation; green, antibiotic target alteration; purple, antibiotic target protection; orange, antibiotic target replacement.

The CARD database, Resfinder, and ISfinder were used to detect different antibiotic resistance genes and ISs in each isolate. The antibiotic resistance genes and ISs detected by WGS are shown in Fig. 3 and 4, respectively, and the resistance mechanisms are shown in Fig. 5. Figure 3 also shows the expected resistance to antibiotic classes based on antibiotic resistance gene presence (purple) and the actual resistance observed by AST (maroon). Clustering based on the prevalence of antibiotic resistance genes and ISs shows significant grouping based on the different sequence types of the isolates. Resfinder (Fig. S1) detected antibiotic resistance genes similar to CARD, with the exception of efflux pumps, which were not detected by Resfinder.

10.1128/mSphere.00725-21.4 FIG S1 Heat map showing the presence or absence of each antibiotic resistance gene as detected by the ResFinder database. Dark green shows the presence of the antibiotic resistance gene, while light green indicates its absence. Download FIG S1, TIF file, 1.8 MB. Copyright © 2021 Jalal et al. 2021 Jalal et al. https://creativecommons.org/licenses/by/4.0/ This content is distributed under the terms of the Creative Commons Attribution 4.0 International license.

Genes encoding three classes of aminoglycoside-modifying enzymes were detected across the isolates—aminoglycoside adenyltransferases (ANT), aminoglycoside acetyltransferases (AAC), and aminoglycoside phosphotransferases (APH). The plasmid carrying *ant-(2″)-1a* was predominant in the ST1 group (55% of the isolates, *n *= 17), whereas the integron-associated *ant(3″)-Ia* was found across different ST groups (in 74% of the isolates, *n *= 23). Other aminoglycoside-modifying enzymes were identified in isolates across all ST groups, such as the integron-associated *aac(3)-Ia* and *aac(6′)-1b ′* and five variants of APHs. The most common APH gene was the plasmid-associated *aph(3′)-VIa*, being detected in 97% (*n *= 30) of the isolates.

β-lactamases are a class of antibiotic-hydrolyzing enzymes that target the β-lactam ring in penicillins, cephalosporins, carbapenems, and monobactams. Genes encoding several classes of β-lactamases were found in isolates across all ST groups. Extended-spectrum class A β-lactamase (ESBL) genes were only found in a few isolates. This class includes *bla*_PER-7_ (3 isolates) and *bla*_TEM_ variants (12 isolates). Genes encoding class B metallo β-lactamases (MBL) (39), *bla*_NDM_, were found in 20 isolates, predominantly belonging to the ST1 group. Genes encoding class C β-lactamases, *bla*_ADC_, were found distributed across different STs. Finally, genes encoding CHDLs, *bla*_OXA_ genes, which represent the major carbapenem resistance mechanism in A. baumannii, were found in all of the isolates. Two subgroups of *bla*_OXA_ genes, intrinsic *bla*_OXA-51-like_ and acquired *bla*_OXA-23-like_, were found in the majority of the isolates. *bla*_OXA-51-like_ (*bla*_OXA-64_, *bla*_OXA-107_, *bla*_OXA-112_, *bla*_OXA-260_, and *bla*_OXA-403_) genes were found in 90% of the isolates, whereas *bla*_OXA-23-like_ (*bla*_OXA-103_, *bla*_OXA-482_, and *bla*_OXA-483_) were found in 97% of the isolates. The presence of IS*Aba1* upstream of *bla*_OXA_ genes strongly contributes to antibiotic resistance by overexpressing the *bla*_OXA_ genes (18, 20, 40, 41). The presence of both IS*Aba1* and IS*Aba125* was observed across all isolates, possibly contributing to the carbapenem resistance of these strains (Fig. 3).

Other antibiotic-modifying enzymes were also detected across isolates of different STs. The chloramphenicol acetyltransferase genes, *catB8* and *catI*, were present in 22.6% of the isolates. The rifampin ADP-ribosyl transferase gene *arr-2* was only found in isolates belonging to ST113. The macrolide phosphotransferase gene *mphE* was present in all isolates except those belonging to ST1. The streptothricin acetyltransferase gene (*SAT-2*) was confined to isolates belonging to ST1.

Genes encoding several classes of efflux pumps that are intrinsic to A. baumannii were detected in the isolates, including resistance-nodulation-division (RND), major facilitator superfamilies (MFS), small multidrug resistance (SMR) family, and multidrug and toxic compound extrusion (MATE) transporter (42). Genes encoding RND family efflux pumps (*adeABC*, *adeFGH*, and *adeIJK*) as well as their regulators (*adeL*, *adeN*, and *adeRS*) were detected in all the isolates. MFS gene *abaQ* was found in all isolates except those belonging to ST1, whereas *cmlA* was predominant in ST113. Other members of the MFS were present in almost all the isolates, such as *abaF*, *amvA*, and *tetAB*. *abeM* and *abeS*, which belong to the MATE and SMR families, respectively, were found in almost all isolates.

The intrinsic A. baumannii genes *sul1* and *sul2*, which confer resistance to sulfonamides, were found in 84% of the isolates, while the integron-associated *dfrA1*, which confers resistance to trimethoprim, was only present in ST1 (55% of the isolates). Two genes were absent from ST1 while present in all other STs—the plasmid-associated *armA*, which provides resistance to aminoglycosides by altering its target 30S ribosomal subunit, and *msrE*, which provides resistance against erythromycin/streptogramin B by protecting its target 50S ribosomal subunit (Fig. 3 and 5).

### Virulence genes in A. baumannii.

The Virulence Factors Database was used to detect virulence factors encoded on both genomic and plasmid DNA in the isolates. As expected for a highly virulent pathogen such as A. baumannii, different classes of virulence genes were detected across all isolates. The gene encoding OmpA, which provides adherence to host cells (43, 44), was detected in all of the isolates. Genes encoding virulence factors that help in biofilm formation, such as the efflux pump AdeFGH (45), the fimbriae CsuABCDE (46), and PNAG polysaccharide biosynthesis gene products PgaABCD (47), were detected in all isolates, except the biofilm-associated protein (*bap*), which was absent in ST1 isolates and the majority of ST113 isolates. Phospholipases (*plc*, *plcD*), which help in host cell lysis (48, 49), were detected in all isolates. Virulence genes responsible for iron uptake through the production of the siderophore acinetobactin (50), such as *barAB*, *basABCFGHI*, *bauBCDEF*, and *entE*, were found in all isolates except *bauA*, which was absent in ST1 isolates and the majority of ST113 isolates. Regulatory genes such as those responsible for quorum sensing (*abaIR*) (51) and the two-component system *bfmRS* (52) were detected in all isolates (Table 1). We found a significant association between the absence of *bauA* and *bap* and recovery in patients with bacteremia (29.4% versus 0%; Table 2), while controlling for colistin sensitivity (22) (*r* = 0.439, *P =* 0.032).

**TABLE 1:** Virulence factors in each isolate

Virulence mechanism		Virulence gene	Pasteur ST [no. of isolates (%)]					All isolates
			ST1	ST2	ST600	ST570	ST113	
Adherence		*ompA*	17/17 (100)	7/7 (100)	2/2 (100)	1/1 (100)	4/4 (100)	31/31 (100)
Biofilm formation								
Efflux pump	* adeF*, *adeG*, *adeH*		17/17 (100)	7/7 (100)	2/2 (100)	1/1 (100)	4/4 (100)	31/31 (100)
Biofilm-associated protein	* bap*		0/17 (0)	7/7 (100)	2/2 (100)	1/1 (100)	1/4 (25)	21/31 (67.7)
Csu fimbrae	* csuA*, *csuA/B*, *csuC*, *csuD*, *csuE*		17/17 (100)	7/7 (100)	2/2 (100)	1/1 (100)	4/4 (100)	31/31 (100%)
Biofilm formation	* pgaA*, *pgaB*, *pgaC*, *pgaD*		17/17 (100)	7/7 (100)	2/2 (100)	1/1 (100)	4/4 (100)	31/31 (100%)
Phospholipase		*plc*, *plcD*	17/17 (100)	7/7 (100)	2/2 (100)	1/1 (100)	4/4 (100)	31/31 (100%)
Acinetobactin production		*barA*, *barB*, *basA*, *basB*, *basC*, *basF*, *basG*, *basH*, *basI*, *basJ*, *entE*	17/17 (100)	7/7 (100)	2/2 (100)	1/1 (100)	4/4 (100)	31/31 (100)
		*bauA*	0/17 (0)	7/7 (100)	2/2 (100)	1/1 (100)	1/4 (25)	21/31 (67.7)
		*bauB*, *bauC*, *bauD*, *bauE*, *bauF*	17/17 (100)	7/7 (100)	2/2 (100)	1/1 (100)	4/4 (100)	31/31 (100)
Regulation								
Quorum sensing	* abaI*, *abaR*		17/17 (100)	7/7 (100)	2/2 (100)	1/1 (100)	4/4 (100)	31/31 (100)
Two-component system	* bfmR*, *bfmS*		17/17 (100)	7/7 (100)	2/2 (100)	1/1 (100)	4/4 (100)	31/31 (100s)

**TABLE 2:** Number of recovered patients with bacteremia

	No. of recovered patients with bacteremia	
	Present (%)	Absent (%)
*bauA/bap* genes	0/8 (0)	5/17 (29.4)
ColR	0/4 (0)	5/21 (23.8)

### Identification of prophage and CRISPR sequences.

**FIG 6:**
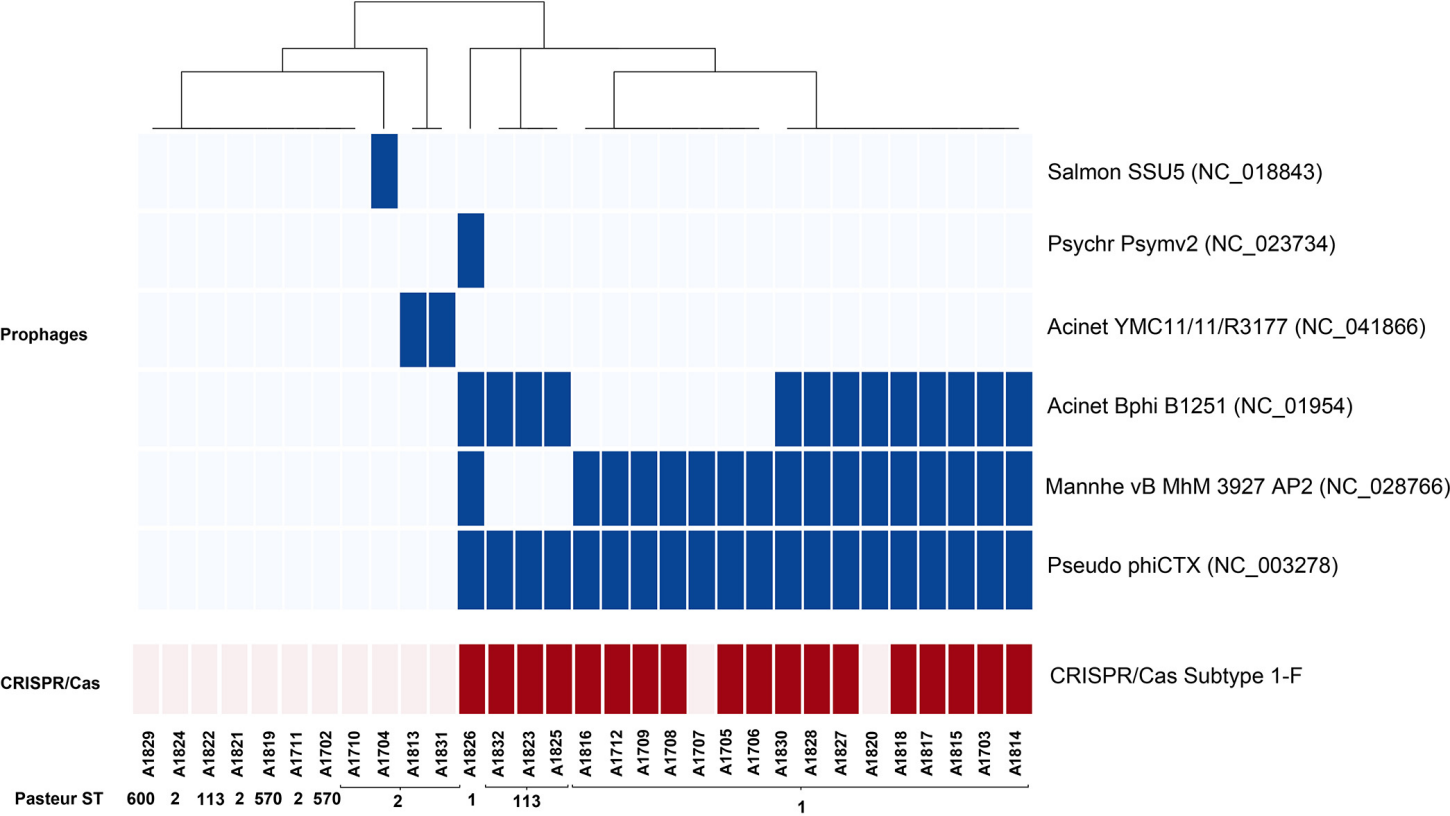
Heat map showing the prophage and CRISPR/Cas sequence distribution among isolates.

The identification of prophage sequences using the PHASTER tool revealed the presence of intact prophages in 77% (*n *= 24) of the isolates. The following six intact prophages were detected among all isolates; Pseudo phiCTX (GenBank accession number NC_003278), Mannhe vB MhM 3927AP2 (NC_028766), Acinet Bphi B1251 (NC_019541), Acinet YMC11/11/R3177 (NC_041866), Psychr Psymv2 (NC_023734), and Salmon SSU5 (NC_018843) (Fig. 6).

Identification of the CRISPR regions using the CRISPR-CasFinder tool has revealed the presence of complete CRISPR-Cas subtype I-F in 18 samples, while 3 samples contain CRISPR sequences with no Cas genes. All of the CRISPR sequences identified were 28-nucleotide (nt)-long identical sequences, GTTCATGGCGGCATACGCCATTTAGAAA or TTTCTAAATGGCGTATGCCGCCATGAAC for the forward (+) or reverse (–) direction, respectively. The CRISPR sequences are interspersed among the same 53 spacers in 15 of the isolates all belonging to ST1 or 98 spacers in 3 of the isolates all belonging to ST113.

The presence of intact CRISPR/Cas sequences was predominant in ST1 and ST113 and was found to strongly correlate with the presence of prophages (*P* = 0.00065), in particular, Pseudo phiCTX (*P* = 0.00001), Mannhe vB MhM 3927AP2 (*P =* 0.00026), and Acinet Bphi B1251 (*P* = 0.0024). The prophages Acinet YMC11/11/R3177, Psychr Psymv2, and Salmon SSU5, on the other hand, were not associated with the presence of CRISPR/Cas.

### Emergence of colistin-resistant strains.

Four out of the 31 A. baumannii isolates (A1712, A1816, A1820, and A1828) were found by AST to be colistin resistant. Isolates A1712 and A1816 were obtained from patients that had colistin-susceptible strains prior to colistin administration, suggesting the development of resistance upon colistin exposure. Few mechanisms of colistin resistance have been previously reported, such as the loss of LPS (28) and, more commonly, the modification of lipid A by the addition of phosphoethanolamine (pEtN), by PmrA and PmrB, altering its charge and thus its interaction with colistin (21, 22, 53–57). Horizontal gene transfer of the Escherichia coli pEtN transferase genes *mcr1* and *mcr4.3* was recently found to contribute to colistin resistance in A. baumannii (58, 59).

The LPS biosynthesis genes, *lpsB*, *lpxA*, *lpxB*, *lpxC*, *lpxD*, *lpxL*, and *lpxM*, were analyzed using *in silico* gene analysis and were found in an unmutated intact form in all of the ColR isolates. No *mcr* genes or associated plasmids were detected during antibiotic resistance gene detection. Mutation analysis was then performed to identify unique mutations that only exist in ColR strains, not in ColS strains (Table 2 and Table S3). Previously reported mutations in *pmrA*, L20F and M12I (53, 54, 60), were found in isolates A1712 and A1828, respectively (Table 3). Two mutations were identified in *lptF*, R30H and N82D, a lipopolysaccharide ABC transporter permease previously implicated with colistin resistance (60). Several new mutations in outer membrane proteins were identified, such as the OmpW and OmpA family proteins, the outer membrane porin OprD, the pilin *pilA*, the multidrug efflux MFS transporter AmvA, TonB-dependent receptor, a LysE family translocator, and a DMT family transporter. Mutations in other proteins, including hypothetical proteins, were also found and are also reported in Table S3.

**TABLE 3:** Unique mutations in colistin-resistant strains compared to colistin-susceptible strains^*a*^

Protein mutation/name	GenBank protein accession no.	Mutations in colistin-resistant strains			
		A1712	A1816	A1820	A1828
*pmrAB* mutations					
DNA-binding response regulator PmrA	WP_000161506.1	L20F (60)			M12I (53, 54, 60)
Outer membrane proteins					
Outer membrane beta-barrel protein, OmpW family	WP_000472952.1	L81F, N82D		L81F, N82D	
OmpA family protein	WP_000777880.1			E146fs	
Outer membrane porin, OprD family	WP_171059678.1			P86T	
TonB-dependent receptor	WP_086242031.1		V443A		
Pilin pilA	WP_000993718.1		A29Q		
LPS export ABC transporter permease LptF (60)	WP_000586912.1	R30H			
ABC transporter permease	WP_000096309.1		H196Y		A41V
Multidrug efflux MFS transporter AmvA	WP_042791420.1			Q294L	
LysE family translocator	WP_024437491.1	G109D		A108L, G109D	A108L, G109D
DMT family transporter	WP_000340904.1			V293fs	

a STOP codon inserted. fs, frameshift.

10.1128/mSphere.00725-21.3 TABLE S3 Additional mutations identified in colistin-resistant strains Table S3, DOCX file, 0.01 MB. Copyright © 2021 Jalal et al. 2021 Jalal et al. https://creativecommons.org/licenses/by/4.0/ This content is distributed under the terms of the Creative Commons Attribution 4.0 International license.

## DISCUSSION

In this study, 31 hospital-acquired MDR A. baumannii isolates were sequenced to identify the antibiotic resistance mechanisms, virulence genes, and other characteristic sequences, such as ISs, phages, and CRISPR/Cas regions. Our results show distinct patterns of distribution of antibiotic resistance and virulence genes with significant clustering of isolates based on their ST. ST1 and ST2, belonging to international clone I and II (ICI and ICII), respectively, are widely spread throughout the world and have been the cause of many hospital outbreaks in several countries (61–63), including Egypt (10, 13). Even though the prevalence of ST1 and ST2 in our study showed wide temporal distribution (Fig. 1), there was a low rate of acquired mutations across isolates belonging to the same clonal complex (Fig. 3 and 5 and Table 1). The distribution of ST113, on the other hand, was confined to late 2018 and showed a distinct pattern of resistance and virulence. Isolates belonging to ST113 did not belong to a previously identified Oxford ST and were not previously reported in Egypt.

CRAB represents a major global health threat, frequently implicated in MDR outbreaks, and was announced as a global first priority for antibiotic development by the WHO in 2017 (64). The majority of antibiotic resistance genes detected in our study are chromosomally encoded, suggesting transposon-dependent gene transfer. One of the few plasmid-associated antibiotic resistance genes detected in our isolates is the aminoglycoside adenyltransferase *ant-(2″)-1a*, which is harbored on plasmids pABLAC2 and pALAC4-2 and shows similar patterns of distribution across ST1. Transposon-dependent gene transfer is supported by the large number of ISs detected in all isolates, in particular, IS*Aba1* and IS*Aba125*, which are frequently associated with β-lactamase genes (16, 17, 41, 65–67). Some insertion sequences were confined to the ST113 group, such as IS*Aba12*, IS*Aca1*, IS*Aba34*, IS*10A*, IS*Aha2*, IS*1007*, IS*1008*, and IS*Acsp2*. Of these, IS*1007*, IS*1008*, IS*Aba34*, and IS*Aha2* were found to be associated with the sulfonamide resistance gene *sul2* (68).

Underlying the wide range of antibiotic resistance exhibited by A. baumannii is the presence of antibiotic efflux pumps. Unlike other antibiotic resistance genes, efflux pumps exhibit substrate promiscuity and thus provide resistance against a large range of antibiotics. Genes encoding RND family efflux pumps are prevalent in A. baumannii (Fig. 3; 69), but their expression is tightly controlled by regulatory genes. The expression of RND antibiotic efflux pumps, *adeABC*, *adeFGH*, and *adeIJK*, is tightly controlled by regulatory genes *adeRS*, *adeL*, and *adeN*, respectively. The most clinically significant efflux pumps are *adeABC*, as they provide resistance to tigecycline, one of the first-line antibiotics used to treat multidrug-resistant pathogens. All of our isolates were susceptible to tigecycline, with the exception of isolates A1829 and A1705, despite similar RND gene profiles among all isolates (Fig. 3). Interruptions in *adeRS* by insertion sequence IS*Aba1* enhance overexpression of the *adeABC* efflux pump and decrease the susceptibility of A. baumannii to tigecycline (70, 71) and thus may account for the tigecycline resistance observed in these two isolates.

Increasing use of colistin has caused the emergence of colistin-resistant A. baumannii. Colistin resistance mechanisms, while detrimental to patient treatment, reduce the fitness and virulence of A. baumannii (72, 73). This makes colistin-resistant A. baumannii less likely to propagate between patients (74) and more likely to develop following a prior colistin exposure. Here, we report two mutations in *pmrA*, which have been previously reported to contribute to colistin resistance (53, 54, 60) but not in Egypt (12). We also report novel mutations in several proteins, including outer membrane proteins, such as OmpA, OprD, and AmvA. These mutations need further *in vitro* studies to understand the effect and mechanism of resistance.

CRAB infections are associated with the highest mortality rates (15 to 35%) among patients with hospital-acquired infections (75–77). Moreover, immune suppression, which is common during cancer therapy, is one of the major factors that negatively affect prognosis, further increasing the fatality from A. baumannii infections (78). The pathogenicity of A. baumannii strongly depends on the arsenal of virulence genes encompassed in its genome. The ability of A. baumannii to survive *in vivo* principally depends on immune evasion, serum resistance, and iron uptake. A subset of our isolates, belonging to ST1 and ST113, showed the absence of *bap* and *bauA* virulence genes, important for biofilm formation and acinetobactin production, respectively. Acinetobactin production, in particular *bauA*, was previously associated with A. baumannii persistence inside and induction of apoptosis in human alveolar epithelial cells, as well as the infection and killing of mice and Galleria mellonella larvae (50). Consistently, we observed a higher recovery rate in patients with bacteremia among isolates lacking *bauA* and *bap*, (29.4% versus 0%), suggesting reduced *in vivo* survival of A. baumannii. This highlights the importance of iron acquisition for A. baumannii virulence and supports the use of iron chelators/mimics as adjunct antibacterial therapy (79–81).

The worldwide spread of MDR pathogens has renewed interest in the development of novel antimicrobial therapeutic options. Developing resistance to colistin, the last resort in antibiotic therapy, limits the antimicrobial therapy options and poses a huge health care crisis. Reduced susceptibility to antibiotics is frequently overcome by using combination antimicrobial therapy. Immunization against A. baumannii provides an attractive alternative for the management of hospital-acquired outbreaks and could be particularly useful for protecting the highly vulnerable pediatric cancer patients. One of the attractive “old” treatment options is the use of bacteriophages. Susceptibility to phage therapy is largely reduced by the presence CRISPR/Cas sequences in the bacterial genome and increased in the presence of *bap* and *ompA* virulence genes (82). Understanding the genetic background of the circulating A. baumannii strains would help decide on the viability of phages as treatment options.

In conclusion, using whole-genome sequencing of MDR A. baumannii from a pediatric cancer hospital, this study revealed the presence of several lineages of A. baumannii circulating in Egypt, including a novel sequence type, ST-2246. Furthermore, through identification of the genetic determinants of multidrug resistance and virulence, we reveal a high rate of horizontal gene transmission of antibiotic resistance genes, in addition to point mutations in outer membrane proteins in individual samples causing colistin resistance. Our results highlight the importance of developing new antimicrobial agents and support the use of iron chelators/mimics as adjunct therapy, as deficient iron acquisition correlates with higher recovery. On the other hand, our results discourage the use of bacteriophage therapy due to the high prevalence of CRISPR/Cas sequences, which negatively affects the response to bacteriophage therapy.

## MATERIALS AND METHODS

### Bacterial isolates.

A total of 31 A. baumannii isolates were collected between October 2016 and December 2018 from hospitalized patients at CCHE 57357. Bacterial species identification was performed in duplicate, using the Vitek mass spectrometry (MS) in vitro diagnostic (IVD) system (bioMérieux; Marcy l’Étoile, France) according to the manufacturer’s instructions. The bacteria were isolated from different sources—blood (*n *= 16), central venous port blood (*n *= 6), bronchioalveolar lavage fluid (*n *= 6), wound (*n *= 1), tissue (*n *= 1), and pleural fluid (*n *= 1).

### Antimicrobial susceptibility testing (AST).

AST was performed using the Vitek 2 AST cards GN222 (bioMérieux SA, Marcy l’Étoile, France) according to the manufacturer’s instructions. Interpretation of results was done according to CLSI guidelines (83).

### DNA extraction.

Single colonies were subcultured in 2 ml Luria-Bertani (LB) medium (Oxoid) and incubated overnight at 37°C with shaking (MaxQ 4000 benchtop shaker; Thermo Fisher Scientific). The bacterial cultures were pelleted by centrifugation for 10 min at 5,000 × *g*. Bacterial DNA extraction was performed using a QIAamp DNA minikit (Qiagen, Germany) following the protocols for bacteria according to the manufacturer’s instructions and stored at −20°C until used for library preparation.

### Library preparation and next-generation sequencing.

Library preparation was performed using the Nextera XT DNA library preparation kit (Illumina, USA). The DNA was prepared, fragmented, and tagged using transposome contained in the Nextera XT kit, and unique adapters were added to each sample to label it. A 12-cycle PCR was performed to amplify the tagmented DNA to add the primers and indices for dual-indexed sequencing of pooled libraries. Samples were then normalized, pooled, and subjected to 300-base paired-end read sequencing using an Illumina MiSeqDx system. The sample preparation and sequencing were performed according to the manufacturer’s protocol.

### Quality control, genome assembly, and alignment.

The generated read pairs were quality filtered, and adapters were removed using fastp (84). The quality of all samples before and after filtration using fastp are summarized in Table S1. After that, the filtered reads were error corrected and merged using SPAdes BayesHammer (85) and PEAR (86), respectively. The merged reads were *de novo* assembled using SPAdes (85). The assessment of the assembled files was carried out with QUAST (87) as shown in Table S2.

10.1128/mSphere.00725-21.1 TABLE S1 The quality of all WGS samples before and after filtration and filtering result using fastp Table S1, DOCX file, 0.02 MB. Copyright © 2021 Jalal et al. 2021 Jalal et al. https://creativecommons.org/licenses/by/4.0/ This content is distributed under the terms of the Creative Commons Attribution 4.0 International license.

10.1128/mSphere.00725-21.2 TABLE S2 The evaluation of all assemblies using QUAST Table S2, DOCX file, 0.01 MB. Copyright © 2021 Jalal et al. 2021 Jalal et al. https://creativecommons.org/licenses/by/4.0/ This content is distributed under the terms of the Creative Commons Attribution 4.0 International license.

### Variant calling, variant effect prediction, and phylogenetic tree.

Previously merged reads were mapped to the A. baumannii reference (GenBank accession number NZ_CP009257.1) using Bowtie 2 (88). Variant identification and filtration were performed using BCFtools (89), SAMtools (90) and VarScan 2 (91). The resulting variants were used to predict the variant effect using SnpEff (92), as well as consensus sequence generation using BCFtools (89). After that, the multiple sequence alignment was done using MAFFT (93), followed by maximum likelihood phylogenetic tree generation using IQ-TREE (94). The best fit model calculation was performed, and GTR+F+I+G4 was the best model. The phylogenetic tree visualization was done using T-REX (95).

### Identification of the resistome, virulome, and mobile genetic elements and multilocus sequence typing (MLST).

**FIG 7:**
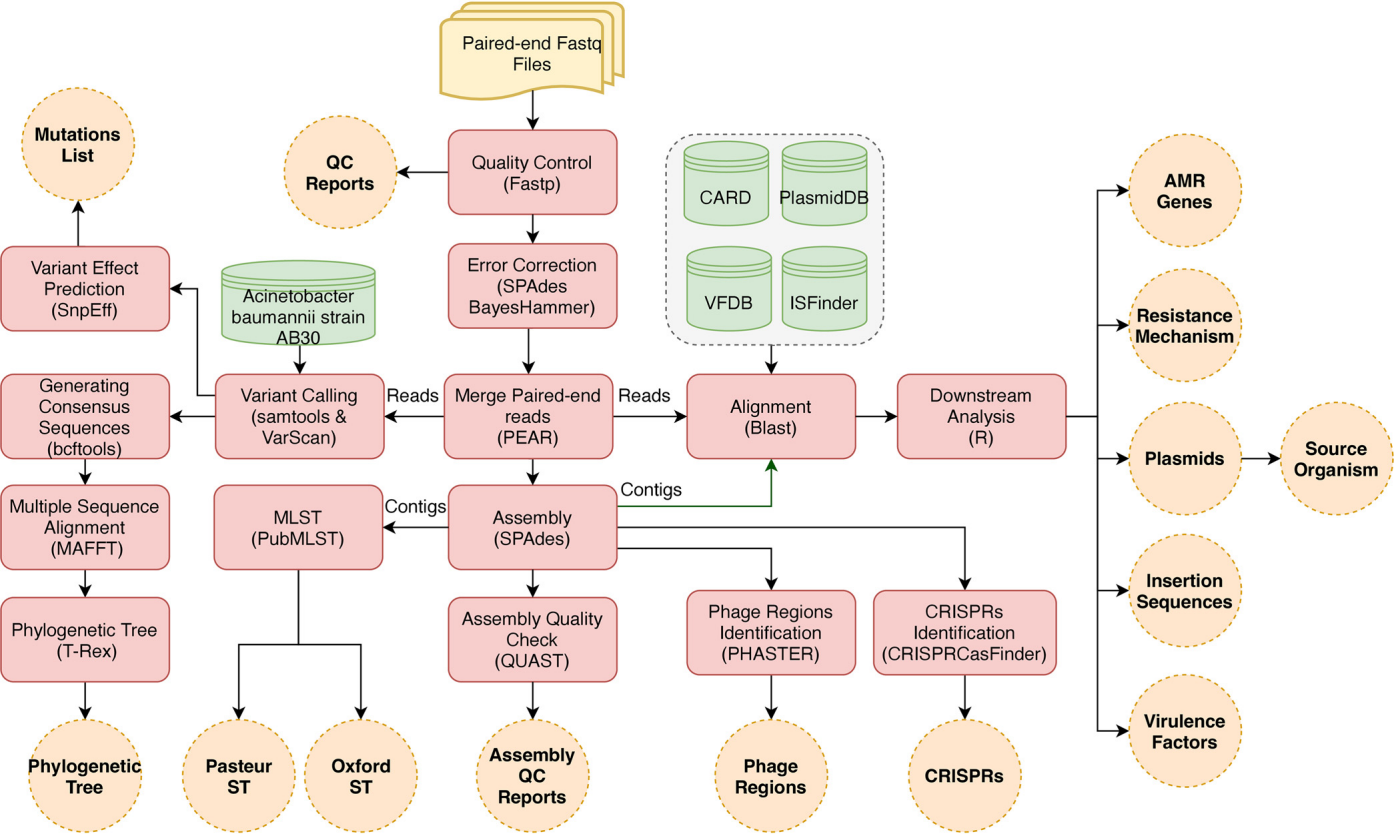
Bioinformatics workflow. The workflow shows the bioinformatics pipeline used to analyze the whole-genome sequencing data generated from Illumina MiSeqDx.

The profiling of antibiotic resistance genes and resistance mechanisms took place by aligning the reads against The Comprehensive Antibiotic Resistance Database (CARD; https://card.mcmaster.ca/) (96). Plasmid-borne virulence factors and insertion sequences were detected by aligning the reads to the Virulence Factors Database (http://www.mgc.ac.cn/VFs/) (97) and ISfinder database (https://github.com/thanhleviet/ISfinder-sequences) (98), respectively. In addition, plasmids were characterized by aligning the contigs of each sample against the database of plasmid sequences (99). All previous alignments were performed using BLAST (100). The bioinformatics pipeline used to analyze and visualize our data is illustrated in Fig. 7.

The identification and visualization of the phage regions and clustered regularly interspaced short palindromic repeats (CRISPR) were performed using PHASTER (https://phaster.ca/) (101) and CRISPRCasFinder (https://crisprcas.i2bc.paris-saclay.fr/) (102), respectively. PubMLST (https://pubmlst.org/) (103) was used to determine the sequence type of each sample by uploading contigs and determining the allelic variation among seven housekeeping genes in the Oxford (*cpn60*, *gyrB*, *gltA*, *gdhB*, *recA*, *gpi*, and *rpoD*) and Pasteur (*cpn60*, *fusA*, *gltA*, *pyrG*, *recA*, *rplB*, and *rpoB*) schemes.

### Downstream analysis and visualization.

The results of the alignments were imported to Rstudio (104) for further analysis. Reads with less than 90% identity or a 1e-4 E value were filtered out. The gene coverage was defined as the percentage of covered bases in each gene. Then, the gene-copy-number was calculated by dividing the number of reads aligned to each gene by its length. We set a cutoff coverage of 75%. Hence, only genes with sequencing reads covering over 75% of the length of the gene were included in any downstream analyses. The other results of the analysis were visualized in heat maps, stacked bar plots, and lollipop charts using the ggplot2 package (https://ggplot2.tidyverse.org/) (105).

### Statistical analysis.

Fisher’s exact test was used to analyze the association between the presence of prophages and intact CRISPR/Cas sequences. Data with a *P* value of <0.05 were considered statistically significant.

### Ethical approval.

Ethical approval was not required, as the isolates were collected as part of routine clinical care and patient data collection followed patient discharge from the hospital and/or death. No additional isolates were collected beyond those obtained from routine clinical care, and no diagnostic or treatment decisions were affected by the outcomes of this study.

### Data availability.

All data generated and analyzed during this study are included in this article and published online on NCBI with the SRA accession number PRJNA688598.
