# Supplementary figures and images for: Deciphering Multidrug-Resistant Acinetobacter baumannii from a Pediatric Cancer Hospital in Egypt
Source: mSphere. 2021 Nov 17;6(6):e00725-21. doi: 10.1128/mSphere.00725-21 (PMC8597740; doi:10.1128/mSphere.00725-21)

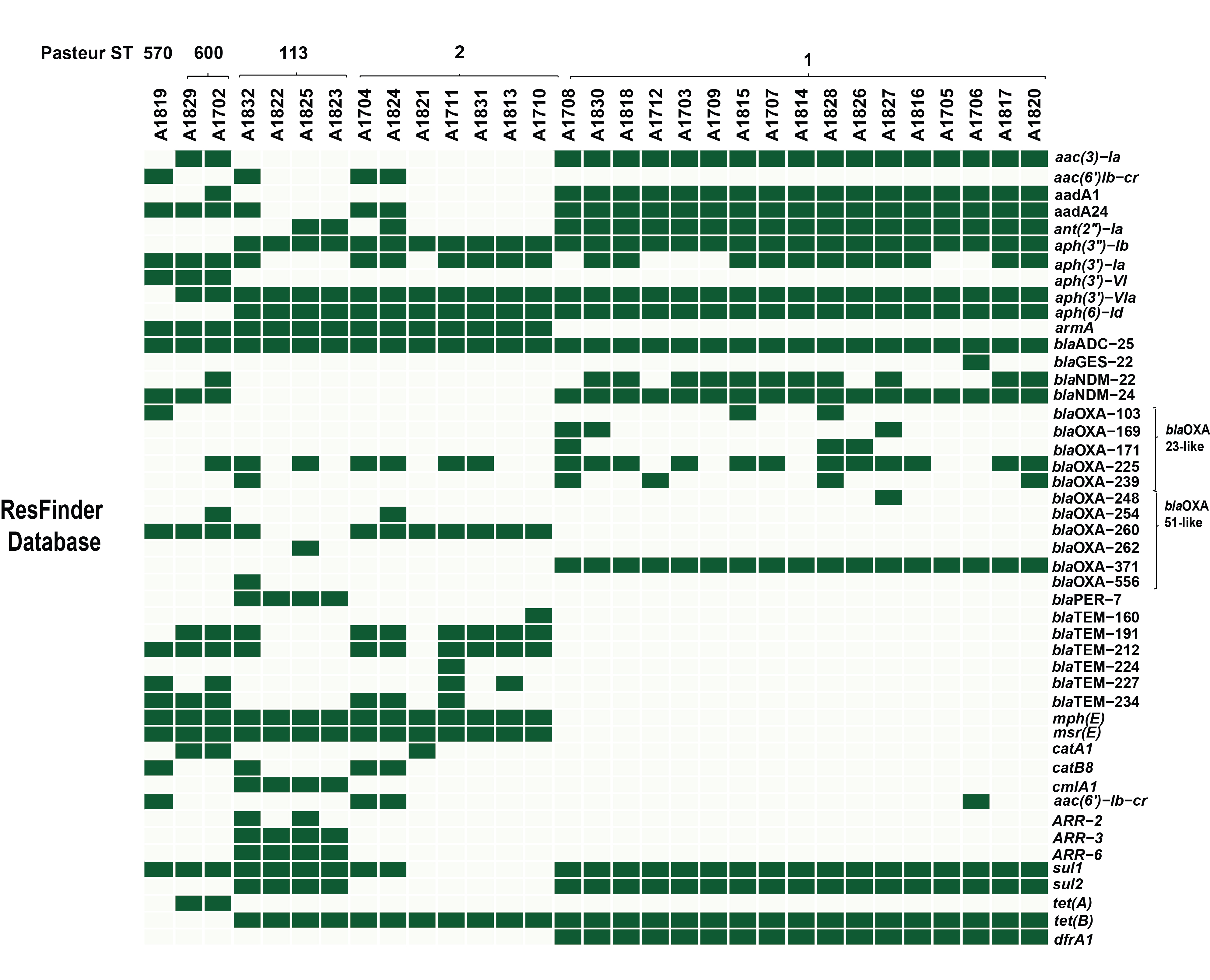

Supplement: FIG S1 [file msphere.00725-21-sf001.tif]
