# Supplementary material for: Deciphering Multidrug-Resistant Acinetobacter baumannii from a Pediatric Cancer Hospital in Egypt
Source: mSphere. 2021 Nov 17;6(6):e00725-21. doi: 10.1128/mSphere.00725-21 (PMC8597740; doi:10.1128/mSphere.00725-21)
Supplement: TABLE S3 [file msphere.00725-21-st003.docx]

## Supplementary Table 3: Additional mutations identified in Colistin Resistant strains

| **Protein mutations** | | **Colistin Resistant strains** | | | |
| --- | --- | --- | --- | --- | --- |
|  |  | **A1712** | **A1816** | **A1820** | **A1828** |
|  |  | MIC = Undetermined | MIC ≥ 8 ug/ul | MIC ≥16 ug/ul | MIC ≥ 4 ug/ul |
| **Protein name** | **Protein accession number** |  |  |  |  |
| elongation factor G fusA | WP_000113824.1 |  |  | V126A |  |
| LLM class flavin-dependent oxidoreductase | WP_024437495.1 |  | A427T | A427T |  |
| ring-hydroxylating dioxygenase ferredoxin reductase family protein | WP_000064951.1 | V210A |  |  |  |
| metal-dependent hydrolase | WP_000626177.1 |  |  |  | L123F |
| AraC family transcriptional regulator | WP_024437386.1 |  |  |  | S62F |
| aspartate aminotransferase family protein | WP_024437390.1 |  | L303P | L303P |  |
| ATP-binding domain-containing protein | WP_024437394.1 |  |  |  | LIEins500 |
| gamma-glutamylputrescine synthetase | WP_000105619.1 |  |  | T443A |  |
| 2-amino-4-hydroxy-6-hydroxymethyldihydropteridine diphosphokinase | WP_000993409.1 |  |  | T130S |  |
| aminopeptidase P family protein | WP_024437196.1 |  | E182K |  |  |
| Ig-like domain repeat protein | WP_042791510.1 | V2006A | V2006A |  |  |
| glycosyltransferase family 25 protein | WP_000760350.1 |  |  |  | T67A |
| aromatic ring-hydroxylating dioxygenase subunit alpha | WP_001100889.1 |  |  | K47T |  |
| DUF4124 domain-containing protein | WP_001054469.1 |  |  | KGins82 |  |
| alpha/beta hydrolase | WP_024437097.1 |  | V97A, S99G |  |  |
| alpha/beta fold hydrolase | WP_024437102.1 |  |  | A36T |  |
| DNA circularization N-terminal domain-containing protein | WP_024437124.1 | L128V |  |  | L128V |
| GTP 3’,8-cyclase MoaA | WP_087737198.1 |  | K214R |  | R178K |
| - **Hypothetical proteins:** | | | | | |
| hypothetical protein | WP_005123832.1 | E61D |  |  |  |
| hypothetical protein | WP_000191722.1 | V231L |  |  |  |
| hypothetical protein | WP_106451083.1 | P126N |  |  |  |
| hypothetical protein | WP_001035103.1 |  |  |  | N33Y |
| hypothetical protein | WP_000209708.1 |  |  | L21F |  |
| hypothetical protein | WP_001255248.1 |  |  | Y119H |  |
| hypothetical protein | WP_000889270.1 | I17L,I18fs |  |  |  |
| hypothetical protein | WP_000981543.1 |  |  | V272E, Y273* |  |

*= STOP codon inserted

FS= frameshift

Ins= aminoacid insertion
