# Supplementary material for: Deciphering Multidrug-Resistant Acinetobacter baumannii from a Pediatric Cancer Hospital in Egypt
Source: mSphere. 2021 Nov 17;6(6):e00725-21. doi: 10.1128/mSphere.00725-21 (PMC8597740; doi:10.1128/mSphere.00725-21)
Supplement: TABLE S1 [file msphere.00725-21-st001.docx]

## Supplementary Table 1: The table shows the quality of all WGS samples before and after filtration and filtering result using Fastp

| **General** | **Samples** | **A1702** | **A1703** | **A1704** | **A1705** | **A1706** | **A1707** | **A1708** | **A1709** | **A1710** | **A1711** |  |
| --- | --- | --- | --- | --- | --- | --- | --- | --- | --- | --- | --- | --- |
|  | **sequencing:** | paired end (301 cycles + 301 cycles) | paired end (301 cycles + 301 cycles) | paired end (301 cycles + 301 cycles) | paired end (301 cycles + 301 cycles) | paired end (301 cycles + 301 cycles) | paired end (301 cycles + 301 cycles) | paired end (301 cycles + 301 cycles) | paired end (301 cycles + 301 cycles) | paired end (301 cycles + 301 cycles) | paired end (301 cycles + 301 cycles) |  |
|  | **mean length before filtering:** | 186bp, 194bp | 188bp, 197bp | 193bp, 203bp | 165bp, 172bp | 166bp, 181bp | 214bp, 227bp | 205bp, 216bp | 219bp, 234bp | 208bp, 218bp | 219bp, 227bp |  |
|  | **mean length after filtering:** | 178bp, 179bp | 181bp, 181bp | 184bp, 185bp | 157bp, 158bp | 156bp, 158bp | 204bp, 206bp | 195bp, 196bp | 207bp, 209bp | 200bp, 201bp | 210bp, 210bp |  |
|  | **duplication rate:** | 1.03% | 0.99% | 0.92% | 1.09% | 1.01% | 0.58% | 0.75% | 0.56% | 0.76% | 0.67% |  |
|  | **Insert size peak:** | 141 | 142 | 122 | 100 | 116 | 177 | 152 | 195 | 184 | 161 |  |
| **Before Filtering** | **total reads:** | 3.181376 M | 3.271186 M | 3.130086 M | 3.758354 M | 3.081460 M | 1.783560 M | 2.111140 M | 1.584514 M | 2.709736 M | 1.980074 M |  |
|  | **total bases:** | 606.496234 M | 632.485961 M | 621.250422 M | 633.695887 M | 536.366285 M | 394.225070 M | 445.199634 M | 359.920554 M | 578.696481 M | 442.043857 M |  |
|  | **Q20 bases:** | 527.290984 M (86.940521%) | 551.659234 M (87.220787%) | 530.909060 M (85.458141%) | 556.238697 M (87.776915%) | 449.546502 M (83.813341%) | 327.232139 M (83.006425%) | 375.855572 M (84.424052%) | 292.155310 M (81.172166%) | 495.969779 M (85.704647%) | 373.437833 M (84.479815%) |  |
|  | **Q30 bases:** | 463.707892 M (76.456846%) | 486.452757 M (76.911234%) | 460.583853 M (74.138196%) | 493.596629 M (77.891720%) | 386.811882 M (72.117113%) | 277.584066 M (70.412586%) | 322.792861 M (72.505195%) | 244.637328 M (67.969813%) | 429.646598 M (74.243859%) | 319.355404 M (72.245185%) |  |
|  | **GC content:** | 42.53% | 42.87% | 42.46% | 42.01% | 43.09% | 42.59% | 41.21% | 41.29% | 42.03% | 41.38% |  |
| **After Filtering** | **total reads:** | 3.034468 M | 3.129750 M | 2.952478 M | 3.607730 M | 2.920288 M | 1.634668 M | 1.960540 M | 1.384216 M | 2.562974 M | 1.848576 M |  |
|  | **total bases:** | 542.547922 M | 568.519057 M | 546.730741 M | 570.293850 M | 460.231621 M | 335.916323 M | 385.015654 M | 288.621127 M | 515.461131 M | 389.373064 M |  |
|  | **Q20 bases:** | 494.977668 M (91.232064%) | 519.607358 M (91.396647%) | 492.572386 M (90.094145%) | 524.605805 M (91.988684%) | 415.305751 M (90.238422%) | 296.529971 M (88.274951%) | 344.276156 M (89.418742%) | 252.959709 M (87.644211%) | 463.133224 M (89.848331%) | 344.894603 M (88.576903%) |  |
|  | **Q30 bases:** | 442.900394 M (81.633414%) | 465.823395 M (81.936285%) | 435.837650 M (79.717056%) | 473.266811 M (82.986483%) | 366.121274 M (79.551525%) | 257.713164 M (76.719453%) | 302.294274 M (78.514801%) | 219.032619 M (75.889323%) | 408.117708 M (79.175263%) | 300.414378 M (77.153354%) |  |
|  | **GC content:** | 42.03% | 42.39% | 41.98% | 41.59% | 42.44% | 41.99% | 40.58% | 40.53% | 41.51% | 40.87% |  |
| **Filtering Results** | **reads passed filters:** | 3.034468 M (95.382250%) | 3.129750 M (95.676308%) | 2.952478 M (94.325779%) | 3.607730 M (95.992288%) | 2.920288 M (94.769622%) | 1.634668 M (91.651977%) | 1.960540 M (92.866413%) | 1.384216 M (87.359026%) | 2.562974 M (94.583900%) | 1.848576 M (93.358935%) |  |
|  | **reads with low quality:** | 91.590000 K (2.878943%) | 84.074000 K (2.570138%) | 114.202000 K (3.648526%) | 97.744000 K (2.600713%) | 122.148000 K (3.963965%) | 103.452000 K (5.800309%) | 102.100000 K (4.836250%) | 159.418000 K (10.061003%) | 82.234000 K (3.034761%) | 74.124000 K (3.743496%) |  |
|  | **reads with too many N:** | 55.262000 K (1.737047%) | 57.328000 K (1.752514%) | 63.354000 K (2.024034%) | 52.812000 K (1.405190%) | 38.992000 K (1.265374%) | 45.410000 K (2.546032%) | 48.434000 K (2.294211%) | 40.856000 K (2.578456%) | 64.486000 K (2.379789%) | 57.288000 K (2.893225%) |  |
|  | **reads too short:** | 56 (0.001760%) | 34 (0.001039%) | 52 (0.001661%) | 68 (0.001809%) | 32 (0.001038%) | 30 (0.001682%) | 66 (0.003126%) | 24 (0.001515%) | 42 (0.001550%) | 86 (0.004343%) |  |
| **General** | **Samples** | **A1712** | **A1813** | **A1814** | **A1815** | **A1816** | **A1817** | **A1818** | **A1819** | **A1820** | **A1821** |  |
|  | **sequencing:** | paired end (301 cycles + 301 cycles) | paired end (301 cycles + 301 cycles) | paired end (301 cycles + 301 cycles) | paired end (301 cycles + 301 cycles) | paired end (301 cycles + 301 cycles) | paired end (301 cycles + 301 cycles) | paired end (301 cycles + 301 cycles) | paired end (301 cycles + 301 cycles) | paired end (301 cycles + 301 cycles) | paired end (301 cycles + 301 cycles) |  |
|  | **mean length before filtering:** | 210bp, 219bp | 189bp, 197bp | 214bp, 228bp | 184bp, 201bp | 192bp, 209bp | 199bp, 222bp | 192bp, 205bp | 188bp, 200bp | 204bp, 218bp | 158bp, 166bp |  |
|  | **mean length after filtering:** | 200bp, 201bp | 181bp, 181bp | 203bp, 205bp | 172bp, 174bp | 180bp, 182bp | 185bp, 188bp | 182bp, 183bp | 179bp, 180bp | 192bp, 194bp | 148bp, 149bp |  |
|  | **duplication rate:** | 0.57% | 0.79% | 0.57% | 0.79% | 0.74% | 0.67% | 0.68% | 0.93% | 0.61% | 0.19% |  |
|  | **Insert size peak:** | 165 | 143 | 157 | 139 | 126 | 138 | 139 | 142 | 144 | 35 |  |
| **Before Filtering** | **total reads:** | 1.748892 M | 2.548992 M | 2.015896 M | 2.939436 M | 2.762776 M | 2.541634 M | 2.412218 M | 2.253588 M | 1.897124 M | 309.042000 K |  |
|  | **total bases:** | 375.946958 M | 492.374854 M | 446.558233 M | 567.177430 M | 556.194838 M | 536.510938 M | 480.411364 M | 438.528245 M | 400.998473 M | 50.306165 M |  |
|  | **Q20 bases:** | 316.521983 M (84.193256%) | 427.903151 M (86.905972%) | 366.026111 M (81.966042%) | 468.129430 M (82.536682%) | 455.419246 M (81.881243%) | 427.743071 M (79.726813%) | 402.133194 M (83.706012%) | 372.383473 M (84.916645%) | 328.108217 M (81.822810%) | 42.304226 M (84.093522%) |  |
|  | **Q30 bases:** | 270.597538 M (71.977584%) | 375.470361 M (76.257014%) | 307.976507 M (68.966707%) | 398.067855 M (70.184008%) | 385.070510 M (69.233025%) | 355.954835 M (66.346240%) | 343.550509 M (71.511737%) | 321.305145 M (73.268974%) | 275.374692 M (68.672255%) | 36.470837 M (72.497749%) |  |
|  | **GC content:** | 41.42% | 41.68% | 42.19% | 41.71% | 42.02% | 41.24% | 41.39% | 40.45% | 40.98% | 39.22% |  |
| **After Filtering** | **total reads:** | 1.622732 M | 2.434098 M | 1.815672 M | 2.692958 M | 2.501640 M | 2.191432 M | 2.240596 M | 2.113146 M | 1.712156 M | 285.042000 K |  |
|  | **total bases:** | 326.697781 M | 442.205070 M | 371.387602 M | 468.113371 M | 454.454763 M | 409.768563 M | 410.239555 M | 380.353516 M | 331.152641 M | 42.452632 M |  |
|  | **Q20 bases:** | 290.036846 M (88.778334%) | 402.448441 M (91.009459%) | 325.769500 M (87.716848%) | 419.271021 M (89.566128%) | 403.996269 M (88.896916%) | 361.304982 M (88.172938%) | 366.893512 M (89.433968%) | 342.964697 M (90.169982%) | 291.415241 M (88.000277%) | 38.373658 M (90.391705%) |  |
|  | **Q30 bases:** | 253.193700 M (77.500894%) | 358.945242 M (81.171670%) | 281.808236 M (75.879818%) | 367.735113 M (78.556849%) | 352.764998 M (77.623787%) | 313.629524 M (76.538210%) | 321.343155 M (78.330612%) | 302.652133 M (79.571273%) | 251.970571 M (76.088951%) | 33.953994 M (79.980893%) |  |
|  | **GC content:** | 40.87% | 41.21% | 41.52% | 40.85% | 41.19% | 40.21% | 40.65% | 39.73% | 40.21% | 38.55% |  |
| **Filtering Results** | **reads passed filters:** | 1.622732 M (92.786290%) | 2.434098 M (95.492571%) | 1.815672 M (90.067742%) | 2.692958 M (91.614786%) | 2.501640 M (90.548057%) | 2.191432 M (86.221384%) | 2.240596 M (92.885303%) | 2.113146 M (93.768071%) | 1.712156 M (90.250084%) | 285.042000 K (92.234065%) |  |
|  | **reads with low quality:** | 80.376000 K (4.595824%) | 67.770000 K (2.658698%) | 147.248000 K (7.304345%) | 203.426000 K (6.920579%) | 214.050000 K (7.747642%) | 307.392000 K (12.094267%) | 126.838000 K (5.258148%) | 100.670000 K (4.467099%) | 142.054000 K (7.487861%) | 19.348000 K (6.260638%) |  |
|  | **reads with too many N:** | 45.740000 K (2.615370%) | 47.062000 K (1.846298%) | 52.912000 K (2.624739%) | 43.012000 K (1.463274%) | 47.046000 K (1.702852%) | 42.762000 K (1.682461%) | 44.688000 K (1.852569%) | 39.646000 K (1.759239%) | 42.852000 K (2.258788%) | 4.646000 K (1.503356%) |  |
|  | **reads too short:** | 44 (0.002516%) | 62 (0.002432%) | 64 (0.003175%) | 40 (0.001361%) | 40 (0.001448%) | 48 (0.001889%) | 96 (0.003980%) | 126 (0.005591%) | 62 (0.003268%) | 6 (0.001941%) |  |
| **General** | **Samples** | **A1822** | **A1823** | **A1824** | **A1825** | **A1826** | **A1827** | **A1828** | **A1829** | **A1830** | **A1831** | **A1832** |
|  | **sequencing:** | paired end (301 cycles + 301 cycles) | paired end (301 cycles + 301 cycles) | paired end (301 cycles + 301 cycles) | paired end (301 cycles + 301 cycles) | paired end (301 cycles + 301 cycles) | paired end (301 cycles + 301 cycles) | paired end (301 cycles + 301 cycles) | paired end (301 cycles + 301 cycles) | paired end (301 cycles + 301 cycles) | paired end (301 cycles + 301 cycles) | paired end (301 cycles + 301 cycles) |
|  | **mean length before filtering:** | 177bp, 189bp | 206bp, 221bp | 186bp, 200bp | 216bp, 233bp | 209bp, 224bp | 211bp, 224bp | 178bp, 188bp | 213bp, 223bp | 190bp, 202bp | 238bp, 250bp | 243bp, 255bp |
|  | **mean length after filtering:** | 164bp, 165bp | 196bp, 198bp | 176bp, 178bp | 204bp, 206bp | 196bp, 198bp | 201bp, 203bp | 170bp, 171bp | 203bp, 204bp | 180bp, 181bp | 228bp, 229bp | 233bp, 235bp |
|  | **duplication rate:** | 0.13% | 0.60% | 0.65% | 0.45% | 0.53% | 0.77% | 0.97% | 0.25% | 0.72% | 0.42% | 0.43% |
|  | **Insert size peak:** | 35 | 163 | 124 | 162 | 158 | 185 | 127 | 140 | 131 | 203 | 222 |
| **Before Filtering** | **total reads:** | 317.766000 K | 2.724918 M | 2.438440 M | 1.652812 M | 1.589710 M | 2.670542 M | 2.470192 M | 612.526000 K | 2.154224 M | 1.767374 M | 2.127834 M |
|  | **total bases:** | 58.384479 M | 583.822636 M | 471.835905 M | 372.193683 M | 345.048691 M | 581.987055 M | 453.402993 M | 133.791063 M | 423.194453 M | 432.423229 M | 531.098887 M |
|  | **Q20 bases:** | 47.541427 M (81.428194%) | 485.763216 M (83.203902%) | 396.779501 M (84.092689%) | 302.107908 M (81.169542%) | 274.894713 M (79.668383%) | 483.210245 M (83.027662%) | 390.862662 M (86.206458%) | 111.398768 M (83.263236%) | 357.895193 M (84.569916%) | 349.932443 M (80.923600%) | 427.993383 M (80.586383%) |
|  | **Q30 bases:** | 40.048546 M (68.594508%) | 414.476162 M (70.993507%) | 341.929587 M (72.467903%) | 253.769405 M (68.182083%) | 226.137853 M (65.537954%) | 410.069011 M (70.460160%) | 342.150319 M (75.462739%) | 94.871248 M (70.910004%) | 309.291676 M (73.085002%) | 290.199467 M (67.110055%) | 353.845868 M (66.625232%) |
|  | **GC content:** | 40.25% | 42.21% | 40.78% | 40.91% | 41.38% | 41.96% | 40.81% | 41.66% | 40.97% | 41.63% | 41.90% |
| **After Filtering** | **total reads:** | 283.170000 K | 2.502974 M | 2.259626 M | 1.444864 M | 1.445758 M | 2.472156 M | 2.343824 M | 561.012000 K | 2.000130 M | 1.558986 M | 1.865562 M |
|  | **total bases:** | 46.677412 M | 495.097475 M | 400.999246 M | 297.301856 M | 285.973333 M | 500.643148 M | 400.055638 M | 114.254776 M | 362.620354 M | 357.124186 M | 438.139015 M |
|  | **Q20 bases:** | 41.414734 M (88.725429%) | 440.354041 M (88.942898%) | 360.756198 M (89.964308%) | 261.561539 M (87.978441%) | 245.725612 M (85.926058%) | 441.492415 M (88.185051%) | 364.340610 M (91.072485%) | 101.055035 M (88.447099%) | 326.537705 M (90.049469%) | 307.979324 M (86.238719%) | 375.416742 M (85.684390%) |
|  | **Q30 bases:** | 36.131987 M (77.407863%) | 385.306307 M (77.824333%) | 318.782301 M (79.496983%) | 227.291803 M (76.451525%) | 207.709046 M (72.632313%) | 383.117728 M (76.525112%) | 325.113828 M (81.267153%) | 88.049578 M (77.064243%) | 288.954707 M (79.685187%) | 262.526043 M (73.511135%) | 318.804196 M (72.763252%) |
|  | **GC content:** | 39.49% | 41.47% | 40.00% | 40.04% | 40.44% | 41.20% | 40.10% | 40.97% | 40.18% | 41.03% | 41.31% |
| **Filtering Results** | **reads passed filters:** | 283.170000 K (89.112743%) | 2.502974 M (91.855021%) | 2.259626 M (92.666869%) | 1.444864 M (87.418533%) | 1.445758 M (90.944764%) | 2.472156 M (92.571321%) | 2.343824 M (94.884284%) | 561.012000 K (91.589908%) | 2.000130 M (92.846891%) | 1.558986 M (88.209174%) | 1.865562 M (87.674226%) |
|  | **reads with low quality:** | 28.972000 K (9.117401%) | 164.028000 K (6.019557%) | 135.684000 K (5.564377%) | 169.210000 K (10.237704%) | 103.364000 K (6.502066%) | 132.526000 K (4.962513%) | 86.610000 K (3.506205%) | 35.526000 K (5.799917%) | 112.894000 K (5.240588%) | 147.330000 K (8.336096%) | 182.432000 K (8.573601%) |
|  | **reads with too many N:** | 5.614000 K (1.766709%) | 57.878000 K (2.124027%) | 43.022000 K (1.764325%) | 38.658000 K (2.338923%) | 40.472000 K (2.545873%) | 65.772000 K (2.462871%) | 39.540000 K (1.600685%) | 15.974000 K (2.607889%) | 41.130000 K (1.909272%) | 61.030000 K (3.453146%) | 79.770000 K (3.748883%) |
|  | **reads too short:** | 10 (0.003147%) | 38 (0.001395%) | 108 (0.004429%) | 80 (0.004840%) | 116 (0.007297%) | 88 (0.003295%) | 218 (0.008825%) | 14 (0.002286%) | 70 (0.003249%) | 28 (0.001584%) | 70 (0.003290%) |
