# Supplementary material for: Deciphering Multidrug-Resistant Acinetobacter baumannii from a Pediatric Cancer Hospital in Egypt
Source: mSphere. 2021 Nov 17;6(6):e00725-21. doi: 10.1128/mSphere.00725-21 (PMC8597740; doi:10.1128/mSphere.00725-21)
Supplement: TABLE S2 [file msphere.00725-21-st002.docx]

## Supplementary Table 2: The table shows the evaluation of all assemblies using QUAST

| **Samples** | **# contigs** | **Largest contig** | **Total length** | **N50** | **N75** | **L50** | **L75** |
| --- | --- | --- | --- | --- | --- | --- | --- |
| **A1702** | 239 | 138464 | 4118463 | 41601 | 19078 | 30 | 67 |
| **A1703** | 205 | 127524 | 3954212 | 42338 | 23860 | 29 | 62 |
| **A1704** | 225 | 136281 | 3996161 | 40364 | 22239 | 30 | 64 |
| **A1705** | 173 | 152428 | 3955189 | 49456 | 28458 | 27 | 53 |
| **A1706** | 352 | 81016 | 3995005 | 22503 | 11844 | 59 | 118 |
| **A1707** | 220 | 152777 | 3957567 | 40088 | 21565 | 32 | 67 |
| **A1708** | 154 | 165287 | 3965057 | 67495 | 30969 | 20 | 42 |
| **A1709** | 182 | 111974 | 3963074 | 45794 | 27547 | 27 | 55 |
| **A1710** | 212 | 196113 | 4006048 | 45872 | 26713 | 25 | 56 |
| **A1711** | 177 | 222381 | 4012889 | 64668 | 37835 | 19 | 39 |
| **A1712** | 153 | 190363 | 4036655 | 66434 | 34395 | 21 | 42 |
| **A1813** | 208 | 180444 | 3979039 | 35167 | 22981 | 31 | 66 |
| **A1814** | 165 | 261152 | 4026808 | 51452 | 29095 | 22 | 48 |
| **A1815** | 194 | 167792 | 4023955 | 41963 | 26589 | 30 | 60 |
| **A1816** | 198 | 160108 | 4030378 | 50839 | 26276 | 26 | 55 |
| **A1817** | 119 | 217409 | 4039020 | 80196 | 50170 | 15 | 31 |
| **A1818** | 88 | 306017 | 3973759 | 114232 | 68531 | 11 | 23 |
| **A1819** | 109 | 262528 | 3857156 | 104934 | 55364 | 14 | 26 |
| **A1820** | 92 | 270193 | 4041768 | 109890 | 68047 | 11 | 23 |
| **A1821** | 1744 | 48119 | 3870664 | 3137 | 1822 | 378 | 783 |
| **A1822** | 1199 | 31939 | 4136308 | 5320 | 3075 | 243 | 498 |
| **A1823** | 180 | 231243 | 4199700 | 54369 | 28062 | 20 | 45 |
| **A1824** | 87 | 257332 | 3970105 | 135171 | 71294 | 12 | 22 |
| **A1825** | 134 | 308887 | 4216008 | 114040 | 54881 | 12 | 25 |
| **A1826** | 89 | 295946 | 4007949 | 160079 | 84138 | 10 | 19 |
| **A1827** | 106 | 245397 | 4033903 | 89804 | 60490 | 14 | 27 |
| **A1828** | 94 | 257014 | 4039570 | 114189 | 67536 | 11 | 23 |
| **A1829** | 447 | 80707 | 4046712 | 19140 | 9441 | 64 | 142 |
| **A1830** | 95 | 490045 | 4041935 | 144429 | 69086 | 9 | 21 |
| **A1831** | 143 | 176712 | 3981500 | 58465 | 33327 | 22 | 44 |
| **A1832** | 559 | 118344 | 4752283 | 33108 | 12846 | 40 | 94 |
